# Family-based association analysis: a fast and efficient method of multivariate association analysis with multiple variants

**DOI:** 10.1186/s12859-015-0484-5

**Published:** 2015-02-15

**Authors:** Sungho Won, Wonji Kim, Sungyoung Lee, Young Lee, Joohon Sung, Taesung Park

**Affiliations:** 1 Department of Public Health Science, Seoul National University, Seoul, Korea; 2 Interdisciplinary Program of Bioinformatics, Seoul National University, Seoul, Korea; 3 Institute of Health and Environment, Seoul National University, Seoul, Korea; 4 The Center for Genome Science, Korea National Institute of Health, KCDC, Osong, Korea; 5 Department of Statistics, Seoul National University, Seoul, Korea

**Keywords:** Family-based association analysis, Multiple variants, Multiple phenotypes

## Abstract

**Background:**

Many disease phenotypes are outcomes of the complicated interplay between multiple genes, and multiple phenotypes are affected by a single or multiple genotypes. Therefore, joint analysis of multiple phenotypes and multiple markers has been considered as an efficient strategy for genome-wide association analysis, and in this work we propose an omnibus family-based association test for the joint analysis of multiple genotypes and multiple phenotypes.

**Results:**

The proposed test can be applied for both quantitative and dichotomous phenotypes, and it is robust under the presence of population substructure, as long as large-scale genomic data is available. Using simulated data, we showed that our method is statistically more efficient than the existing methods, and the practical relevance is illustrated by application of the approach to obesity-related phenotypes.

**Conclusions:**

The proposed method may be more statistically efficient than the existing methods. The application was developed in C++ and is available at the following URL: http://healthstat.snu.ac.kr/software/mfqls/.

## Background

During the last decade, more than a hundred genome-wide association studies (GWAS) have been initiated, and GWAS have been successful in identifying many susceptibility loci involved in human disease. However, phenotypic variance explained by significant findings has often been small, even for most heritable phenotypes [1,2]. For example, SNPs significantly associated with human height in GWAS involving tens of thousands of subjects explain only about 5% of the phenotypic variance [3]. Various reasons for the so-called missing heritability have been provided [2], but the effect-size distribution for many phenotypes [4] reveals that further investigation of an efficient strategy for genetic association analysis remains necessary.

It has been found that analysis with secondary phenotypes [5-9] reduces false negative findings, and several different methods, such as the linear mixed model [9] and combining of p-values [7], have been proposed. The most efficient approach of multiple phenotypes depends on the unknown disease model between multiple phenotypes and genotypes. For instance, if multiple genes have a causal effect on multiple phenotypes, and the genotype-phenotype models are multidimensional, multivariate analyses are often expected to be most efficient [7]. In such a case, if the marginal effects of genotypes on multiple phenotypes are separately tested, multiple p-values for each marginal effect need to be adjusted with multiple comparison correction methods [10-12], and for a large number of p-values, the chance to identify the disease susceptibility loci becomes smaller. However, joint analysis of multiple phenotypes is much less affected by multiple comparison issues, and is thought to improve power. Furthermore, the presence of linkage disequilibrium (LD) between markers reveals the benefit of multi-marker association analysis [13,14]. For instance, two-marker genome-wide association analysis can sometimes be more efficient than one-marker analysis, if the large-scale genetic information is sufficiently dense [15-17]. Therefore in this report, we focus on the joint analysis of multiple phenotypes and genotypes.

The family-based design has been considered to be an important strategy in genetic association analysis. However the parameter estimations for the analysis of family data is numerically complicated, and few methods other than the linear mixed model for quantitative phenotypes have been available for family-based samples. In particular, FBAT statistics [18], based on the within-family component, has been extended for the joint analysis of multiple phenotypes and genotype [19-21]. Given the nature of FBAT statistics, they are robust against the population substructure and can be combined with rank-based p-values [22,23] based on the between-family component in a robust way [24]. However, even though this approach provides global robustness against population substructure, the phenotypic information is only partially utilized and the loss of power can be substantial if the number of founders is large.

In this report, we propose a new statistical method for the joint analysis of multiple phenotypes and genotypes with family-based samples. Our method can be utilized for both quantitative and dichotomous phenotypes, and is robust against the population substructure if the correlation matrix between individuals can be estimated from large-scale genetic data. The proposed method consists of two steps. First, phenotypes are adjusted with the offset based on the best linear unbiased predictor (BLUP) [25] or disease prevalence. Second adjusted phenotypes are utilized for statistical inference. Using extensive simulations, we showed that our method is statistically more efficient than existing methods, and its computational simplicity makes possible large-scale genome-wide association analysis. The proposed method was applied to the joint analysis of obesity-related phenotypes with the healthy twin study, Korea (HTK) and our significant results illustrate the practical value of the proposed method.

## Methods

### Notations and the disease model

The genetic association between M variants and *Q* phenotypes is considered. We assume that there are n families and ni individuals in family i. If we denote the sample size by *N*, *N* is equal to ${\displaystyle {\sum}_{i=1}^n{n}_i}$. We let *x*_*ijm*_ and *y*_*ijq*_ denote the coded genotype of individual j in family i at variant m and the qth phenotype respectively, where *m = 1*, …, *M* and *q = 1*, …, *Q*. We let

$$ {\mathbf{X}}^m=\left(\begin{array}{c}\hfill {x}_{11m}\hfill \\ {}\hfill \vdots \hfill \\ {}\hfill {x}_{n{n}_nm}\hfill \end{array}\right),\ \mathbf{X}=\left(\begin{array}{c}\hfill {\mathbf{X}}^1\hfill \\ {}\hfill \vdots \hfill \\ {}\hfill {\mathbf{X}}^M\hfill \end{array}\right),\ {\mathbf{Y}}^q=\left(\begin{array}{c}\hfill {y}_{11q}\hfill \\ {}\hfill \vdots \hfill \\ {}\hfill {y}_{n{n}_nq}\hfill \end{array}\right),\ \mathrm{and}\ \mathbf{Y}=\left(\begin{array}{c}\hfill {\mathbf{Y}}^1\hfill \\ {}\hfill \vdots \hfill \\ {}\hfill {\mathbf{Y}}^Q\hfill \end{array}\right). $$

Here X is a *N* × *M* matrix and **Y** is a *N* × *Q* matrix. We also define

$$ {\mathbf{X}}_{ij}=\left(\begin{array}{c}\hfill {x}_{ij1}\hfill \\ {}\hfill \vdots \hfill \\ {}\hfill {x}_{ijM}\hfill \end{array}\right),\ \mathrm{and}\ {\mathbf{Y}}_{ij}=\left(\begin{array}{c}\hfill {y}_{ij1}\hfill \\ {}\hfill \vdots \hfill \\ {}\hfill {y}_{ijQ}\hfill \end{array}\right). $$

We assume that covariate column vector, **Z** *ij*, which affects the phenotype, is observed for individual *j* in family *i*, and the intercept is included in **Z** *ij*. We let

$$ \mathbf{Z}=\left(\begin{array}{c}\hfill {\mathbf{Z}}_{11}^t\hfill \\ {}\hfill \vdots \hfill \\ {}\hfill {\mathbf{Z}}_{n{n}_n}^t\hfill \end{array}\right). $$

In addition, we assume that *b*_*ijq*_ is a random effect for an additive polygenic effect for the *q*th phenotype and the variable *e*_*ijq*_ is a random error. We let

$$ {\mathbf{B}}^q=\left(\begin{array}{c}\hfill {b}_{11q}\hfill \\ {}\hfill \vdots \hfill \\ {}\hfill {b}_{n{n}_nq}\hfill \end{array}\right),\ \mathbf{B}=\left(\begin{array}{c}\hfill {\mathbf{B}}^1\hfill \\ {}\hfill \vdots \hfill \\ {}\hfill {\mathbf{B}}^Q\hfill \end{array}\right),\ {\mathbf{E}}^q=\left(\begin{array}{c}\hfill {e}_{11q}\hfill \\ {}\hfill \vdots \hfill \\ {}\hfill {e}_{n{n}_nq}\hfill \end{array}\right)\ \mathrm{and}\ \mathbf{E}=\left(\begin{array}{c}\hfill {\mathbf{E}}^1\hfill \\ {}\hfill \vdots \hfill \\ {}\hfill {\mathbf{E}}^Q\hfill \end{array}\right). $$

Covariances between individuals are explained by the random effect *b*_*ijq*_, and the variance-covariance matrix for **B** ^q^ can be parameterized by the function of kinship coefficient matrix **Ф**. If we let *π*_*ij,i'j'*_ be the kinship coefficient between individual i in family *j* and individual *i'* in family *j'*, and *d*_*ij*_ be the inbreeding coefficient for individual *j* in family *i*, **Ф**_i_ is denoted by

$$ \left(\begin{array}{cccc}\hfill 1+{d}_{11}\hfill & \hfill 2{\pi}_{11,12}\hfill & \hfill 2{\pi}_{11,13}\hfill & \hfill \cdots \hfill \\ {}\hfill 2{\pi}_{11,12}\hfill & \hfill 1+{d}_{12}\hfill & \hfill 2{\pi}_{12,13}\hfill & \hfill \cdots \hfill \\ {}\hfill 2{\pi}_{11,13}\hfill & \hfill 2{\pi}_{12,13}\hfill & \hfill 1+{d}_{13}\hfill & \hfill \ddots \hfill \\ {}\hfill \vdots \hfill & \hfill \vdots \hfill & \hfill \ddots \hfill & \hfill \ddots \hfill \end{array}\right). $$

and we let

$$ \boldsymbol{\Phi} =\left(\begin{array}{ccc}\hfill {\boldsymbol{\Phi}}_1\hfill & \hfill 0\hfill & \hfill \cdots \hfill \\ {}\hfill 0\hfill & \hfill {\boldsymbol{\Phi}}_2\hfill & \hfill \ddots \hfill \\ {}\hfill \vdots \hfill & \hfill \ddots \hfill & \hfill \ddots \hfill \end{array}\right). $$

Under the presence of population substructure, **Ф** should be replaced with the genetic relationship matrix estimated with large-scale genetic data to provide the robustness of the proposed method [26,27]. However the robustness of proposed method depends on the accuracy of the estimated genetic relationship matrix, and if the level of population substructure depends on the genomic location, the proposed method is not valid [23,28]. In such a case, transmission disequilibrium tests based on Mendelian transmission [18,29] are unique choices robust against the population substructure.

### Quasi-likelihood for association analysis

If we let the effect of variant m on phenotype *q* be *β*_*mq*_, the null and alternative hypotheses are

$$ {H}_0:{\beta}_{11}={\beta}_{12}=\cdots ={\beta}_{MQ}=0\kern0.5em \mathrm{v}\mathrm{s}\kern0.75em {H}_1:\kern0.5em \mathrm{not}\kern0.5em {H}_0. $$

Either prospective or retrospective analysis for this hypothesis testing can be selected depending on the sampling scheme. While prospective analysis assumes that phenotypes are response variable and compares the phenotype distributions between each genotype group, retrospective analysis assumed that individuals were selected based on their phenotypes, and compares genotype distributions between affected and unaffected individuals. In particular large numbers of genotypes enables the estimation of genotypic correlations between individuals, and analysis robust against nonnormality of phenotypes can be conducted with retrospective analysis. As a result, we focus on the retrospective analysis which compares genotype frequencies according to disease phenotypes. When comparing the genotype distribution, it has been shown that the statistical efficiency of the test statistic can be improved by adjusting phenotype [30], and we introduce the offset μ_ijq_ for qth phenotype of individual j in family i at variant m to improve the efficiency of the proposed score test. We set

$$ {\boldsymbol{\upmu}}_{ij}=\left(\begin{array}{c}\hfill {\mu}_{ij1}\hfill \\ {}\hfill \vdots \hfill \\ {}\hfill {\mu}_{ijQ}\hfill \end{array}\right),\ \boldsymbol{\upmu} ={\left(\begin{array}{ccc}\hfill {\boldsymbol{\upmu}}_{11}\hfill & \hfill \cdots \hfill & \hfill {\boldsymbol{\upmu}}_{n{n}_n}\hfill \end{array}\right)}^t,\ {\mathbf{T}}_{ij}={\mathbf{Y}}_{ij}-{\boldsymbol{\upmu}}_{ij},\ \mathbf{T}=\mathbf{Y}-\boldsymbol{\upmu} . $$

For any positive integer w, we let **1**_*w*_ be the *w* × 1 column vector that consisted of 1 and **I**_w_ be the w × w identity matrix. We denoted an MAF of variant m in unaffected individuals by *p*_*m*_, and **p** = (*p*_1_, … , *pM*)^t^. We assumed [31] that for a constant *γ*_*m,q*_,

$$ E\left({\mathbf{X}}^m\Big|{\mathbf{T}}^q\right)=2{p}_m{\mathbf{1}}_N+{\gamma}_{m,q}{\mathbf{T}}^q, $$

where 0 < 2 *p*_*m*_ + *γ*_*m,q*_ < 1. If we let **V** be the working correlation matrix for **X** ^m^, the score for a variant m can be defined by

$$ {\mathbf{T}}^t{\mathbf{V}}^{-1}\left({\mathbf{X}}^m-E\left({\mathbf{X}}^m\right)\right). $$

Here **V** and **μ** were incorporated to generalize the quasi-likelihood score function and can be estimated by maximizing the efficiency of the score statistics. Their incorporation is a main difference from the assumptions for WQLS and MQLS statistics [31,32]. The most efficient choices of them makes the proposed score test equivalent to MQLS statistic [33], and we extend this approach to the joint analysis of multiple phenotypes and genotypes. If we let ⊗ indicate the Kronecker product, the quasi-likelihood score corresponding to the null hypothesis is

$$ \mathbf{S}=\mathrm{v}\mathrm{e}\mathrm{c}\left({\mathbf{T}}^t{\mathbf{V}}^{-1}\left(\mathbf{X}-E\left(\mathbf{X}\right)\right)\right)=\mathrm{v}\mathrm{e}\mathrm{c}\left({\mathbf{T}}^t{\mathbf{V}}^{-1}\left(\mathbf{X}-\mathbf{p}\otimes {\mathbf{1}}_N\right)\right). $$

If we let e_*ij*_ be an N × 1 vector where the $\left(j+{\displaystyle {\sum}_{i=1}^{j-1}{n}_i}\right)$ th element is 1 and the others are 0,

$$ \mathbf{T}={\displaystyle \sum_{i,j}{\mathbf{e}}_{ij}{{\mathbf{T}}_{ij}}^t}. $$

Therefore,

$$ \begin{array}{c}\mathbf{S}={\displaystyle \sum_{i,j}\mathrm{v}\mathrm{e}\mathrm{c}\left({\mathbf{T}}_{ij}{{\mathbf{e}}_{ij}}^t{\mathbf{V}}^{-1}\left(\mathbf{X}-\mathbf{p}\otimes {\mathbf{1}}_N\right)\right)}\\ {}={\displaystyle \sum_{i,j}\left\{{\mathbf{I}}_M\otimes \left({\mathbf{T}}_{ij}{{\mathbf{e}}_{ij}}^t\right)\right\}\mathrm{v}\mathrm{e}\mathrm{c}\left({\mathbf{V}}^{-1}\left(\mathbf{X}-\mathbf{p}\otimes {\mathbf{1}}_N\right)\right)}.\end{array} $$

Thus if we define

$$ {\mathbf{S}}_{ij}=\left\{{\mathbf{I}}_M\otimes \left({\mathbf{T}}_{ij}{{\mathbf{e}}_{ij}}^t\right)\right\}\mathrm{v}\mathrm{e}\mathrm{c}\left({\mathbf{V}}^{-1}\left(\mathbf{X}-\mathbf{p}\otimes {\mathbf{1}}_N\right)\right), $$

we have

$$ \mathbf{S}={\displaystyle \sum_{ij}{\mathbf{S}}_{ij}}. $$

### Efficient choices of μ and V

Statistical efficiency depends on the choices of **V** and **μ** [31,33,34], and the optimal choices have been provided by maximizing the non-centrality parameters under the alternative hypothesis (see Won and Lange [33] for detailed information). For **V**, the identity matrix maximizes the statistical efficiency of the quasi-likelihood [33], and we consider it for **V**. The most efficient choice of **μ** may be related with the sampling scheme, and either BLUP or the prevalence were shown to be the most efficient [33], depending on the sampling scheme. If families are randomly selected, BLUP was shown to be the most efficient for both dichotomous and quantitative phenotypes [33], and if families with a large number of affected family members are selectively utilized for association analysis, it was recommended that prevalence was used for dichotomous phenotypes [31,33]. In this report, we focus on randomly selected families, and we incorporate BLUP from the linear mixed model for **μ**. The linear mixed model [35] for quantitative phenotype is given by

1 $$ {\mathbf{Y}}^q=\mathbf{Z}{\boldsymbol{\upalpha}}_q+{\displaystyle \sum_{m=1}^M{\mathbf{X}}^m{\beta}_{mq}}+{\mathbf{B}}^q+{\mathbf{E}}^q,\ \mathrm{v}\mathrm{e}\mathrm{c}\left(\mathbf{B}\right)\sim MVN\left(\mathbf{0},\boldsymbol{\Phi} \otimes {\Sigma}_{\mathbf{B}}\right) $$

and

$$ {\mathbf{E}}^q \sim MVN\left(\mathbf{0},{\sigma}_{\mathbf{E},q}^2{\mathbf{I}}_N\right),\ {\mathbf{E}}^q\hbox{'}\mathrm{s}:\mathrm{indep}. $$

Here, we denote the qth diagonal element for **Σ**_**B**_ by ${\sigma}_{\mathbf{B},q}^2$. Several algorithms to estimate variance parameters such as **Σ**_**B**_ and ${\sigma}_{\mathbf{E},q}^2$ for linear mixed model exist [36-38], and the average information method [36] has often been recommended because of its computational efficiency. If we denote the estimates for ${\sigma}_{\mathbf{B},q}^2$ and ${\sigma}_{\mathbf{E},q}^2$ by ${\widehat{\sigma}}_{\mathbf{B},q}^2$ and ${\widehat{\sigma}}_{\mathbf{E},q}^2$ under the null hypothesis respectively, and

$$ \begin{array}{c}{\mathbf{H}}^q={\widehat{\sigma}}_{\mathbf{B},q}^2\boldsymbol{\Phi} +{\widehat{\sigma}}_{\mathbf{E},q}^2{\mathbf{I}}_N,\ {\mathbf{P}}^q\\ {}={\left({\mathbf{H}}^q\right)}^{-1}-{\left({\mathbf{H}}^q\right)}^{-1}\mathbf{Z}{\left({\mathbf{Z}}^t{\left({H}^q\right)}^{-1}\mathbf{Z}\right)}^{-1}{\mathbf{Z}}^t{\left({\mathbf{H}}^q\right)}^{-1},\end{array} $$

then incorporation of BLUP as offset makes

$$ \begin{array}{l}{\mathbf{T}}^q={\mathbf{Y}}^q-{\widehat{\boldsymbol{\upmu}}}^q=\left({\mathbf{I}}_N-\mathbf{Z}{\left({\mathbf{Z}}^t{\left({\mathbf{H}}^q\right)}^{-1}\mathbf{Z}\right)}^{-1}{\mathbf{Z}}^t{\left({\mathbf{H}}^q\right)}^{-1}-{\widehat{\sigma}}_{1q}^2\boldsymbol{\Phi} {\mathbf{P}}^q\right){\mathbf{Y}}^q,\\ {}\ \mathbf{T}=\left(\begin{array}{ccc}\hfill {\mathbf{T}}^1\hfill & \hfill \cdots \hfill & \hfill {\mathbf{T}}^Q\hfill \end{array}\right).\end{array} $$

For a dichotomous phenotype, the generalized linear mixed model [39] might be considered as an appropriate approach but the generalized linear mixed models cannot be directly optimized. Approximations to avoid numerical integration sometimes lead to serious bias [40,41], and Crowder [42,43] showed that the choice of a linear mixed model for dichotomous phenotypes is reasonable in this context. Therefore we consider the dichotomous phenotypes as quantitative phenotypes, and **T** ^q^ estimated by the same way for quantitative phenotypes was recommended for dichotomous phenotypes when individuals were randomly selected [33]. Therefore, for randomly selected families, we utilize the identity matrix for **V** and BLUP for **μ** for both quantitative and dichotomous.

### Quasi-likelihood maximum estimator for minor allele frequencies

We denote var(**X**_ij_) by **Ψ** and we assume that

$$ \operatorname{cov}\left({\mathbf{X}}_{ij},{\mathbf{X}}_{i\hbox{'}j\hbox{'}}\right)\approx 2{\pi}_{ij,i\hbox{'}j\hbox{'}}\operatorname{var}\left({\mathbf{X}}_{ij}\right)=2{\pi}_{ij,i\hbox{'}j\hbox{'}}\boldsymbol{\Psi} . $$

Then we can have

$$ \operatorname{var}\left(vec\left(\mathbf{X}\right)\right)\Big)=\boldsymbol{\Psi} \otimes \boldsymbol{\Phi} . $$

**Ψ** was estimated with a sample variance covariance matrix, and we found that this choice usually works well. Therefore the marginal quasi-likelihood score function for **p** is

$$ U\left(\mathbf{p}\right)={\left({\mathbf{I}}_M\otimes {\mathbf{1}}_N\right)}^t{\left(\boldsymbol{\Psi} \otimes \boldsymbol{\Phi} \right)}^{-1}\left\{\mathrm{v}\mathrm{e}\mathrm{c}\left(\mathbf{X}\right)-\mathbf{p}\otimes {\mathbf{1}}_N\right\}, $$

and without any knowledge about **Ψ**, the quasi maximum likelihood estimates for **p** can be calculated by

$$ \widehat{\mathbf{p}}={\left\{{\left({\mathbf{1}}_N^t{\boldsymbol{\Phi}}^{-1}{\mathbf{1}}_N\right)}^{-1}{\mathbf{1}}_N^t{\boldsymbol{\Phi}}^{-1}\mathbf{X}\right\}}^t. $$

The quasi-likelihood maximum estimator for **p** is equivalent to the best linear unbiased estimator. We can simply assume that

$$ \mathrm{v}\mathrm{e}\mathrm{c}\left(\mathbf{X}\right)=\left({\mathbf{I}}_M\otimes {\mathbf{1}}_N\right)\mathbf{p}+\mathbf{e},\kern0.5em E\left(\mathbf{e}\right)=0, \operatorname {var}\left(\mathbf{e}\right)=\boldsymbol{\Psi} \otimes \boldsymbol{\Phi} . $$

Therefore Gauss-Markov theorem indicates that the best linear unbiased estimator for **p** is

$$ {\left({\left({\mathbf{I}}_M\otimes {\mathbf{1}}_N\right)}^t{\left(\boldsymbol{\Psi} \otimes \boldsymbol{\Phi} \right)}^{-1}\left({\mathbf{I}}_M\otimes {\mathbf{1}}_N\right)\right)}^{-1}{\left({\mathbf{I}}_M\otimes {\mathbf{1}}_N\right)}^t{\left(\boldsymbol{\Psi} \otimes \boldsymbol{\Phi} \right)}^{-1}\mathrm{v}\mathrm{e}\mathrm{c}\left(\mathbf{X}\right) $$

$$ =\mathrm{v}\mathrm{e}\mathrm{c}\left\{{\left({\mathbf{1}}_N^t{\boldsymbol{\Phi}}^{-1}{\mathbf{1}}_N\right)}^{-1}{\mathbf{1}}_N^t{\boldsymbol{\Phi}}^{-1}\mathbf{X}\right\}. $$

### Family-based multivariate association test

If we let $\mathbf{A} = {\boldsymbol{\Phi}}^{-1}-{\boldsymbol{\Phi}}^{-1}{\mathbf{1}}_N{\left({\mathbf{1}}_N^t{\boldsymbol{\Phi}}^{-1}{\mathbf{1}}_N\right)}^{-1}{\mathbf{1}}_N^t{\boldsymbol{\Phi}}^{-1}$ and utilize the proposed choices of **V** and **μ** and the quasi likelihood maximum estimator for **p**, **S**_ij_ becomes

$$ {\mathbf{S}}_{ij}=\left\{{\mathbf{I}}_M\otimes \left({\mathbf{T}}_{ij}{{\mathbf{e}}_{ij}}^t\right)\right\}\mathrm{v}\mathrm{e}\mathrm{c}\left(\boldsymbol{\Phi} \mathbf{A}\mathbf{X}\right)=\mathrm{v}\mathrm{e}\mathrm{c}\left({\mathbf{T}}_{ij}{{\mathbf{e}}_{ij}}^t\boldsymbol{\Phi} \mathbf{A}\mathbf{X}\right) $$

and our score is

$$ \mathbf{S}={\displaystyle \sum_{ij}{\mathbf{S}}_{ij}}=\mathrm{v}\mathrm{e}\mathrm{c}\left({\displaystyle \sum_{ij}{\mathbf{T}}_{ij}{{\mathbf{e}}_{ij}}^t\boldsymbol{\Phi} \mathbf{A}\mathbf{X}}\right)=\mathrm{v}\mathrm{e}\mathrm{c}\left({\mathbf{T}}^t\boldsymbol{\Phi} \mathbf{A}\mathbf{X}\right). $$

For the statistic based on quasi-likelihood score, we can calculate the covariance of **S**_*ij*_ and **S**_*i'j'*_ as follows:

$$ \begin{array}{l}\operatorname{cov}\left({\mathbf{S}}_{ij},{\mathbf{S}}_{i\hbox{'}j\hbox{'}}\right)=\operatorname{cov}\left(\mathrm{v}\mathrm{e}\mathrm{c}\left({\mathbf{T}}_{ij}{\mathbf{e}}_{ij}^t\boldsymbol{\Phi} \mathbf{A}\mathbf{X}\right),\mathrm{v}\mathrm{e}\mathrm{c}\left({\mathbf{T}}_{i\hbox{'}j\hbox{'}}{\mathbf{e}}_{i\hbox{'}j\hbox{'}}^t\boldsymbol{\Phi} \mathbf{A}\mathbf{X}\right)\right)\\ {}\kern4em =\left({\mathbf{I}}_M\otimes \left({\mathbf{T}}_{ij}{\mathbf{e}}_{ij}^t\boldsymbol{\Phi} \mathbf{A}\right)\right)\operatorname{var}\left(\mathrm{v}\mathrm{e}\mathrm{c}\left(\mathbf{X}\right)\right)\left({\mathbf{I}}_M\otimes \left(\mathbf{A}\boldsymbol{\Phi } {\mathbf{e}}_{i\hbox{'}j\hbox{'}}{\mathbf{T}}_{i\hbox{'}j\hbox{'}}^t\right)\right)\\ {}\kern4em =\boldsymbol{\Psi} \otimes \left({\mathbf{T}}_{ij}{\mathbf{e}}_{ij}^t\boldsymbol{\Phi} \mathbf{A}\boldsymbol{\Phi } {\mathbf{e}}_{i\hbox{'}j\hbox{'}}{\mathbf{T}}_{i\hbox{'}j\hbox{'}}^t\right).\end{array} $$

Therefore, var(S) is

$$ \begin{array}{c}\operatorname{var}\left(\mathbf{S}\right)={\displaystyle \sum_{i,j,i\hbox{'},j\hbox{'}}\operatorname{cov}\left({\mathbf{S}}_{ij},{\mathbf{S}}_{i\hbox{'}j\hbox{'}}\right)}\\ {}=\boldsymbol{\Psi} \otimes \left(\left({\displaystyle \sum_{i,j}{\mathbf{T}}_{ij}{\mathbf{e}}_{ij}^t}\right)\boldsymbol{\Phi} \mathbf{A}\boldsymbol{\Phi } \left({\displaystyle \sum_{i\hbox{'},j\hbox{'}}{\mathbf{T}}_{i\hbox{'}j\hbox{'}}{\mathbf{e}}_{i\hbox{'}j\hbox{'}}^t}\right)\right)\\ {}=\boldsymbol{\Psi} \otimes \left({\mathbf{T}}^t\boldsymbol{\Phi} \mathbf{A}\boldsymbol{\Phi } \mathbf{T}\right),\end{array} $$

and we have

$$ {\mathbf{S}}^t\operatorname{var}{\left(\mathbf{S}\right)}^{-1}\mathbf{S}\sim {\chi}^2\left(df=MQ\right)\kern0.5em \mathrm{under}\kern0.5em {H}_0. $$

The proposed statistic will be denoted as MFQLS in the remainder of this report.

### Utilizing individuals with incomplete information

Individuals with missing genotypes and nonmissing phenotypes, or vice versa, can be utilized for genetic association analysis. For individuals with missing phenotypes and nonmissing genotypes, tijq are assumed to be 0 and these individuals are utilized for the proposed analysis. These individuals are informative only for enhancing the accuracy of the estimated variance–covariance matrix of genotypes **Ψ**. For individuals with missing genotypes and nonmissing phenotypes, the missing genotypes can be replaced with the conditional expectations for the association analysis [44]. We let the superscripts *U* and *O* indicate individuals with missing genotypes and individuals with nonmissing genotypes, respectively. We assume that *N*_*O*_ (*N*_*U*_) indicates the numbers of individuals with nonmissing (missing) genotypes, and in a similar way we define

$$ {\mathbf{Y}}^{*}=\left(\begin{array}{c}\hfill {\mathbf{Y}}^O\hfill \\ {}\hfill {\mathbf{Y}}^U\hfill \end{array}\right),\ \mathrm{and}\ {\boldsymbol{\Phi}}^{*}=\left(\begin{array}{cc}\hfill {\boldsymbol{\Phi}}^{OO}\hfill & \hfill {\boldsymbol{\Phi}}^{OU}\hfill \\ {}\hfill {\boldsymbol{\Phi}}^{UO}\hfill & \hfill {\boldsymbol{\Phi}}^{UU}\hfill \end{array}\right). $$

Then, if we denote the minor allele frequency for variant *m* by *p*_*m*_, the conditional mean vector of the missing genotypes for multiple variants is

$$ 2{p}_m{\mathbf{1}}_{N_U}+{\boldsymbol{\Phi}}^{UO}{\left({\boldsymbol{\Phi}}^{OO}\right)}^{-1}\left({\mathbf{X}}^O-2{p}_m{\mathbf{1}}_{N_O}\right) $$

and the incorporation of best linear unbiased estimator [45] to pm makes it

$$ \begin{array}{l}E\left({\mathbf{X}}^U\Big|{\mathbf{X}}^O\right)=\left\{{\mathbf{1}}_{N^U}{\left({\mathbf{1}}_{N^O}^t{\left({\boldsymbol{\Phi}}^{OO}\right)}^{-1}{\mathbf{1}}_{N^O}\right)}^{-1}{\mathbf{1}}_{N^O}^t{\left({\boldsymbol{\Phi}}^{OO}\right)}^{-1}\right.\\ {}\kern1.25em \left.+{\boldsymbol{\Phi}}^{OU}\left({\left({\boldsymbol{\Phi}}^{OO}\right)}^{-1}-{\left({\boldsymbol{\Phi}}^{OO}\right)}^{-1}{\mathbf{1}}_{N^O}{\left({\mathbf{1}}_{N^O}^t{\left({\boldsymbol{\Phi}}^{OO}\right)}^{-1}{\mathbf{1}}_{N^O}\right)}^{-1}{\mathbf{1}}_{N^O}^t{\left({\boldsymbol{\Phi}}^{OO}\right)}^{-1}\right)\right\}{\mathbf{X}}^O.\end{array} $$

This is an extension of the conditional expectation for a single variant [44]. Therefore, if ${\mathbf{A}}^{*}={\left({\boldsymbol{\Phi}}^{*}\right)}^{-1}-{\left({\boldsymbol{\Phi}}^{*}\right)}^{-1}{\mathbf{1}}_N{\left({\mathbf{1}}_N^t{\left({\boldsymbol{\Phi}}^{*}\right)}^{-1}{\mathbf{1}}_N\right)}^{-1}{\mathbf{1}}_N^t{\left({\boldsymbol{\Phi}}^{*}\right)}^{-1}$, the proposed score and its variance, respectively, become

$$ \begin{array}{l}\kern3.5em {\mathbf{S}}^{*}=\mathrm{v}\mathrm{e}\mathrm{c}\left(\mathbf{W}{\left(\begin{array}{c}\hfill {\mathbf{T}}^O\hfill \\ {}\hfill {\mathbf{T}}^U\hfill \end{array}\right)}^t\left(\begin{array}{c}\hfill {\boldsymbol{\Phi}}^{OO}\hfill \\ {}\hfill {\boldsymbol{\Phi}}^{UO}\hfill \end{array}\right){\mathbf{A}}^{*}\mathbf{X}\right),\\ {}\operatorname{var}\left({\mathbf{S}}^{*}\right)=\boldsymbol{\Psi} \otimes \left(\mathbf{W}{\left(\begin{array}{c}\hfill {\mathbf{T}}^O\hfill \\ {}\hfill {\mathbf{T}}^U\hfill \end{array}\right)}^t\left(\begin{array}{c}\hfill {\boldsymbol{\Phi}}^{OO}\hfill \\ {}\hfill {\boldsymbol{\Phi}}^{UO}\hfill \end{array}\right){\mathbf{A}}^{*}{\left(\begin{array}{c}\hfill {\boldsymbol{\Phi}}^{OO}\hfill \\ {}\hfill {\boldsymbol{\Phi}}^{UO}\hfill \end{array}\right)}^t\left(\begin{array}{c}\hfill {\mathbf{T}}^O\hfill \\ {}\hfill {\mathbf{T}}^U\hfill \end{array}\right)\mathbf{W}\right)\end{array} $$

so that

$$ {{\mathbf{S}}^{*}}^t\operatorname{var}{\left({\mathbf{S}}^{*}\right)}^{-1}{\mathbf{S}}^{*}\sim {\chi}^2\left(df=MQ\right)\kern0.5em \mathrm{under}\kern0.5em {H}_0. $$

### The simulation model

**Figure 1:**
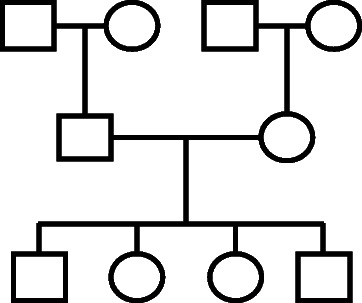
**Extended family used in our simulations.**

In our simulation studies, we considered large families with 10 subjects that extended over three generations (see Figure 1). We assumed the existence of two disease susceptibility loci, and that minor (major) alleles for both loci were denoted by *A*(*a*) and *B*(*b*), respectively. If we denote the minor allele frequencies as *p*_*A*_ and *p*_*B*_, and the linkage disequilibrium between these two loci by *d*, the haplotype frequencies for *AB*, *Ab*, *aB*, and *ab* were calculated by

$$ \begin{array}{l}{p}_{AB}={p}_A{p}_B+d,\ {p}_{Ab}={p}_A\left(1-{p}_B\right)-d,\\ {}\ {p}_{aB}=\left(1-{p}_A\right){p}_B-d,\ \mathrm{and}\ {p}_{ab}=\left(1-{p}_A\right)\left(1-{p}_B\right)+d.\end{array} $$

In our simulation, Lewontin’s *D*' [46] was assumed to be 0 or 0.5. Genotypes were assumed to be in Hardy–Weinberg equilibrium and founders’ genotypes were generated by multinomial distributions defined by genotype frequencies. The non-founders’ genotypes were obtained by simulated Mendelian transmissions from their parents, and we assumed that there was no recombination between two loci.

The quantitative phenotypes were defined by summing the phenotypic mean, polygenic effect, main genetic effect, and random error. We assumed that *Q* = 2 and 5, and denoted the phenotypic means for *Q* phenotypes by α_1_, …, and α_Q_. The genetic effect at variant m for phenotype q was generated by the product of βmq and the number of disease alleles. Under the null hypothesis, the genetic effect size parameters *β*_*mq*_ were set to 0. The polygenic effects B for *Q* phenotypes for each founder was independently generated from MVN(0,Σ_B_), and the average of maternal and paternal polygenic effects was combined with values independently sampled from MVN(0, 0.5Σ_B_) for the polygenic effects of offspring. The random errors for *Q* phenotypes were assumed to be independent and were independently sampled from MVN(0,σ^E^ ^,q^ ^2^ **I**_*N*_). We assume that if *Q* = 2,

$$ {\boldsymbol{\Sigma}}_{\mathbf{B}}=\left(\begin{array}{cc}\hfill 1\hfill & \hfill \rho \sqrt{2}\hfill \\ {}\hfill \rho \sqrt{2}\hfill & \hfill 2\hfill \end{array}\right),{\sigma}_{\mathbf{E},1}^2=1,{\sigma}_{\mathbf{E},2}^2=2 $$

and if Q = 5, they were

$$ \begin{array}{l}{\boldsymbol{\Sigma}}_{\mathbf{B}}=\left(\begin{array}{ccccc}\hfill 1\hfill & \hfill \rho \hfill & \hfill \sqrt{2}\rho \hfill & \hfill \sqrt{2}\rho \hfill & \hfill \sqrt{2}\rho \hfill \\ {}\hfill \rho \hfill & \hfill 1\hfill & \hfill \sqrt{2}\rho \hfill & \hfill \sqrt{2}\rho \hfill & \hfill \sqrt{2}\rho \hfill \\ {}\hfill \sqrt{2}\rho \hfill & \hfill \sqrt{2}\rho \hfill & \hfill 2\hfill & \hfill 2\rho \hfill & \hfill 2\rho \hfill \\ {}\hfill \sqrt{2}\rho \hfill & \hfill \sqrt{2}\rho \hfill & \hfill 2\rho \hfill & \hfill 2\hfill & \hfill 2\rho \hfill \\ {}\hfill \sqrt{2}\rho \hfill & \hfill \sqrt{2}\rho \hfill & \hfill 2\rho \hfill & \hfill 2\rho \hfill & \hfill 2\hfill \end{array}\right),\\ {}{\sigma}_{\mathbf{E},1}^2=1,{\sigma}_{\mathbf{E},2}^2=2,{\sigma}_{\mathbf{E},3}^2=3,{\sigma}_{\mathbf{E},4}^2=4,{\sigma}_{\mathbf{E},5}^2=5.\end{array} $$

Here ρ indicates the correlation between different phenotypes.

Furthermore, the robustness of the proposed statistic in the presence of population substructure was evaluated with simulated data. We assumed that there were two subpopulations and each founder was assigned to one of the two subpopulations with 0.5 probability. Means of *Q* phenotypes in both populations differed by 0.2. The amounts of linkage disequilibrium for both populations were assumed to be same and the allele frequencies for each marker in two subpopulations were generated by the Balding–Nichols model [47]. The allele frequencies, *q*_*A*_ and *q*_*B*_, in an ancestral population was generated from U(0.1, 0.4) and if we let FST be the fixation index by Wright [48], the marker allele frequencies for the two subpopulations were independently sampled from the beta distributions (*p*_*k*_(1 – FST)/FST, (1– *p*_*k*_)(1 – FST)/FST). The value for Wright’s FST was assumed to be 0.01, and 0.05.

Last, the simulations of the dichotomous phenotypes were performed using the liability threshold model. Once the quantitative phenotypes with polygenic effect and random error were generated, they were transformed to being affected if quantitative phenotypes are larger than the threshold, but to unaffected when not. The threshold was chosen to preserve the assumed prevalence. We assumed that prevalence was 0.1 and 0.2 if *Q* = 2, and it was 0.1, 0.1, 0.2, 0.2, and 0.3 if *Q* = 5. The statistical validity of the proposed method for dichotomous phenotypes was also evaluated under the presence of population substructure. Genotypes and liability scores were generated under the same model as used for the quantitative traits with the Balding–Nichols model, and liabilities for each individual were transformed to either being affected or unaffected, respectively.

## Results

### Evaluation of the proposed statistical approach using simulated data

**Figure 2:**
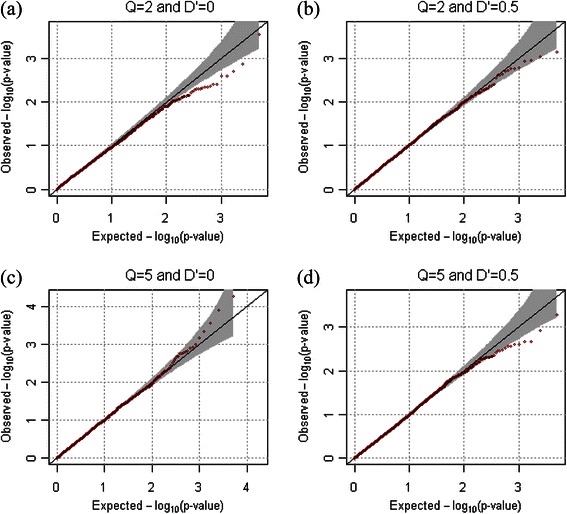
**QQ-plots for quantitative phenotypes in the absence of population substructure.** QQ-plots were generated from results of 10,000 replicates for quantitative phenotypes. We assumed that the number of markers were 2, and that their minor allele frequencies were generated as *U*(0.1, 0.5). *ρ* was assumed to be 0.2. **(a)** *Q*=2 and *D'*=0, **(b)** *Q*=2 and *D'*=0.5, **(c)** *Q*=5 and *D'*=0, and **(d)** *Q*=5 and *D'*=0.5 were assumed respectively.

**Figure 3:**
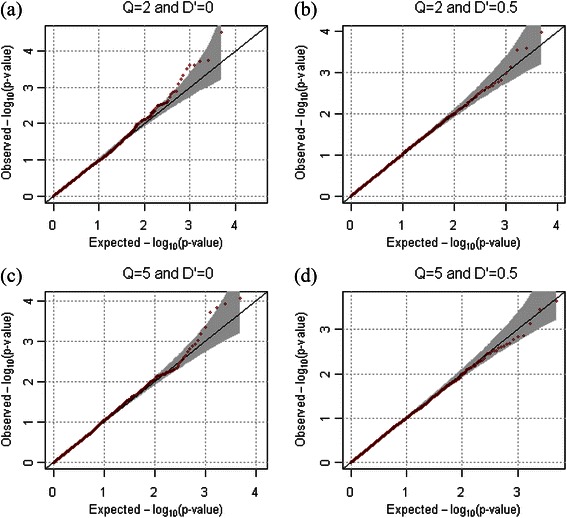
**QQ-plots for dichotomous phenotypes in the absence of population substructure.** QQ-plots were generated from results of 10,000 replicates for quantitative phenotypes. We assumed that the number of markers was 2, and that their minor allele frequencies were generated as *U*(0.1, 0.5). *ρ* was assumed to be 0.2. **(a)** *Q*=2 and *D'*=0, **(b)** *Q*=2 and *D'*=0.5, **(c)** *Q*=5 and *D'*=0, and **(d)** *Q*=5 and *D'*=0.5 were assumed respectively.

For the evaluation of statistical validity, the empirical type-1 error estimates for extended families were calculated at the various significance levels from 10,000 replicates for both dichotomous and quantitative phenotypes. One hundred extended families were generated in each replicate, and we assume that ρ = 0.2. Table 1 shows that the empirical type-1 error rates always preserve the 0.005, 0.01, and 0.05 nominal significance levels for both quantitative and dichotomous phenotypes. The quantile quantile (QQ) plots in Figures 2 and 3 also confirmed the overall validity of our statistical approach for both dichotomous and quantitative phenotypes.

**Table 1:** **Empirical type-I error estimates in the absence of population substructure**

			***α***		
**TYPE**	***Q***	***D*** **'**	**0.005**	**0.01**	**0.05**
Quantitative	2	0	0.0056	0.0105	0.0481
	2	0.5	0.0043	0.0091	0.0482
	5	0	0.0059	0.0115	0.0506
	5	0.5	0.0044	0.0103	0.0526
Dichotomous	2	0	0.0038	0.0088	0.0455
	2	0.5	0.0039	0.0095	0.0502
	5	0	0.0041	0.0083	0.0509
	5	0.5	0.0056	0.0098	0.0501

The empirical type-I errors were estimated with 10,000 replicates at several significance levels. We assumed that the number of markers is two, and that their minor allele frequencies were generated as U(0.1, 0.4). ρ was assumed to be 0.2.

For comparison of power with existing methods, the empirical power estimates were calculated from 2,000 replicates at the 0.005 significance level for quantitative and dichotomous phenotypes. We assumed that ρ were 0.2 and 0.5. For the proposed method, results from different choices of **V** and **μ** were compared, and they were with an omnibus family-based association test (MFBAT) [21]. We let diag(var(**Y** ^1^), …, var(**Y** ^*Q*^)) be the block diagonal matrix that consists of submatrices, var(**Y** ^1^), …, and var(**Y** ^Q^). Then it is a *NQ* × *NQ* dimensional matrix. If diag(var(**Y** ^1^), …, var(**Y** ^Q^)) and BLUP are utilized for **V** and **μ**, respectively, the proposed method for quantitative phenotypes becomes an extension of the mixed-model association score test on related individuals (MASTOR) [9] for the joint analysis of multiple phenotypes and multiple genotypes. For dichotomous phenotypes, if **I**_*NM*_ and the prevalence are utilized for **V** and **μ**, respectively, our score is an extension of the more powerful quasi-likelihood score test (MQLS) [27,31] for the joint analysis of multiple phenotypes and multiple genotypes. Therefore, they will be denoted as MMASTOR and MMQLS in the remainder of this report.

Table 2 shows that MFQLS are always most efficient for both quantitative and dichotomous phenotypes, and it is followed by MMASTOR for quantitative traits, and by MMQLS for dichotomous traits. Even though MFBAT is always least efficient, this method is globally robust to population substructure, and thus MFBAT is still preferred in some scenarios, such as candidate gene analysis. In addition, our results show that the power improvement for each method is proportional to *Q* and *D'*, but inversely related with *ρ*. This result is reminiscent of the analysis of repeated measures, even though results may vary depending on the situation. For the analysis of repeated measurements, it has been shown that power improvement is proportionally related with the number of observations for each individual, but inversely related with the correlation between different measurements [49]. This may be because the larger *D'* leads to reduced standard deviation of the statistics, while the larger *ρ* may induce sample size reduction.

**Table 2:** **Empirical power estimates in the absence of population substructure**

	***ρ***	***Q***	***D*** **'**	**MMASTOR**	**MFBAT**	***MFQLS***
Quantitative phenotypes	0.2	2	0	0.5180	0.2025	0.5830
		2	0.5	0.7235	0.3750	0.7805
		5	0	0.7800	0.3855	0.7870
		5	0.5	0.9200	0.6430	0.9240
	0.5	2	0	0.4915	0.1655	0.5400
		2	0.5	0.6785	0.3340	0.7505
		5	0	0.7015	0.3020	0.7350
		5	0.5	0.8725	0.5405	0.8885
Dichotomous phenotypes	*ρ*	*Q*	*D*'	MMQLS	MFBAT	*MFQLS*
	0.2	2	0	0.2015	0.0530	0.2340
		2	0.5	0.3050	0.1070	0.3470
		5	0	0.3205	0.0995	0.3710
		5	0.5	0.6215	0.2460	0.6660
	0.5	2	0	0.1795	0.0535	0.2130
		2	0.5	0.2945	0.0915	0.3270
		5	0	0.2670	0.0910	0.3085
		5	0.5	0.5200	0.2130	0.5900

The empirical power was estimated using 2,000 replicates at the 0.005 significance level. We assumed that the number of markers was two, and that their minor allele frequencies were 0.2.

### Evaluation with simulated data in the presence of population substructure

**Figure 4:**
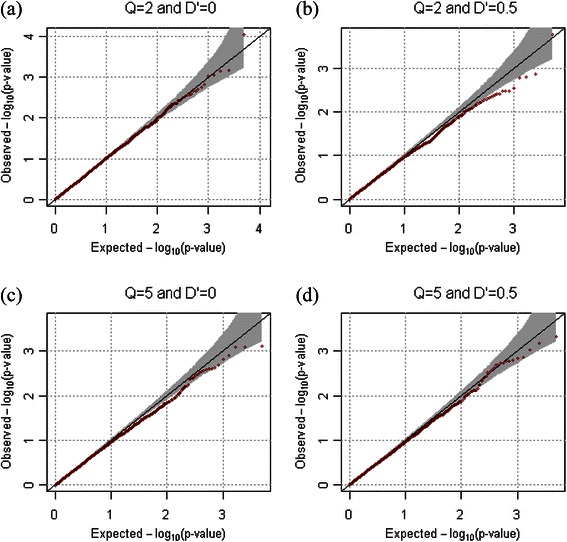
**QQ-plots for quantitative phenotypes in the presence of population substructure.** QQ-plots were generated from results of 10,000 replicates for quantitative phenotypes. We assumed that the number of markers was 2, and that their minor allele frequencies were generated as *U*(0.1, 0.5). *ρ* was assumed to be 0.2, and Wright’s *F*_*ST*_ was assumed to be 0.01. **(a)** *Q*=2 and *D'*=0, **(b)** *Q*=2 and *D'*=0.5, **(c)** *Q*=5 and *D'*=0, and **(d)** *Q*=5 and *D'*=0.5 were assumed respectively.

**Figure 5:**
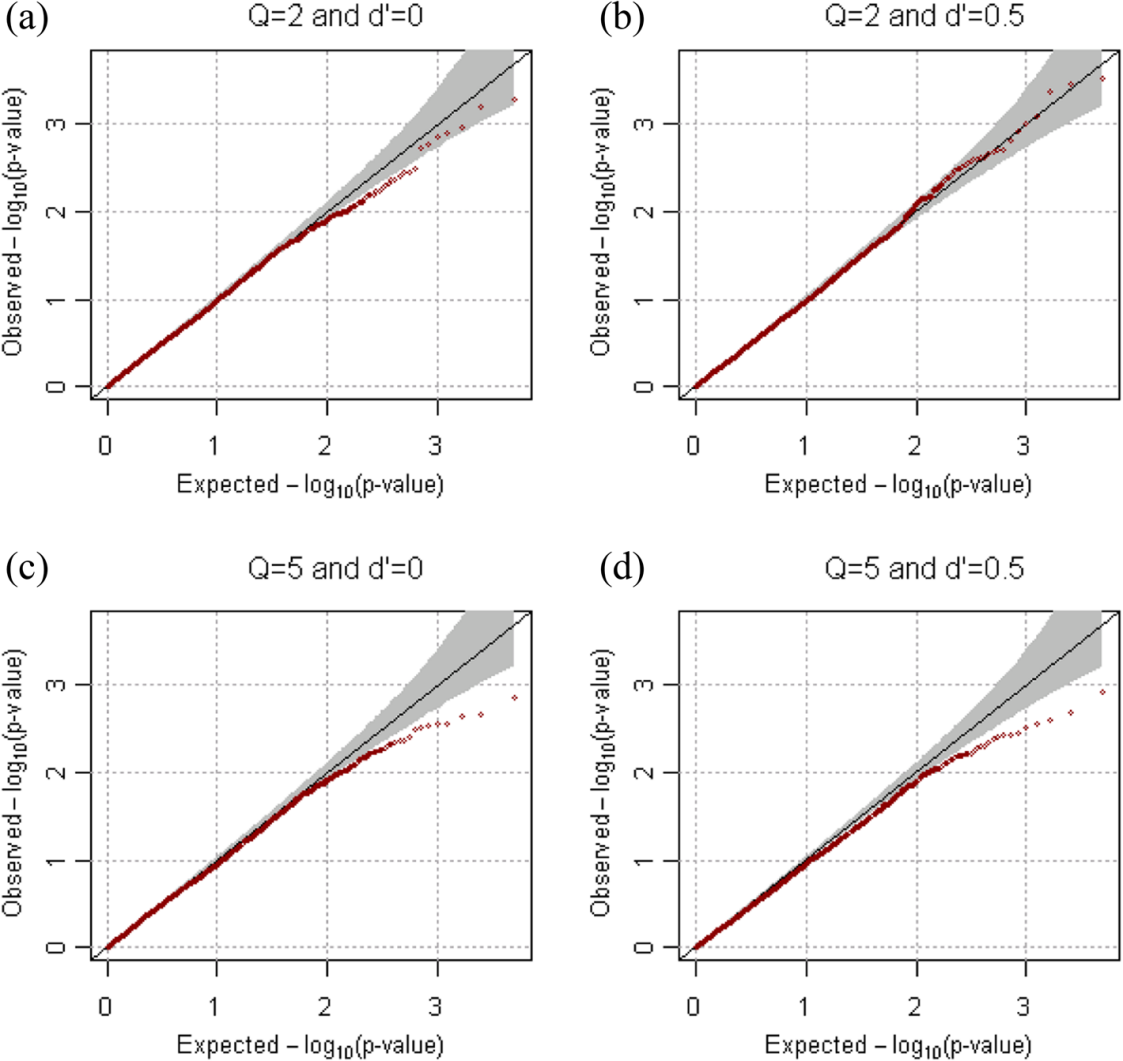
**QQ-plots for dichotomous phenotypes in the presence of population substructure.** QQ-plots were generated from results of 10,000 replicates for quantitative phenotypes. We assumed that the number of markers were 2, and that their minor allele frequencies were generated as *U*(0.1, 0.5). *ρ* was assumed to be 0.2, and Wright’s *F*_*ST*_ was assumed to be 0.01. **(a)** *Q*=2 and *D'*=0, **(b)** *Q*=2 and *D'*=0.5, **(c)** *Q*=5 and *D'*=0, and **(d)** *Q*=5 and *D'*=0.5 were assumed respectively.

The proposed methods for both dichotomous and quantitative phenotypes were evaluated in the presence of population substructure. Wright’s F_ST_ indicates the level of population substructure and we assumed that FST = 0.01 and 0.05. Robustness of the proposed method to population substructure is provided if the genetic relationship matrix is estimated with large-scale genetic information and replace the kinship coefficient matrix [27]. In our simulation studies, we generated 100,000 common variants of which minor allele frequencies were larger than 0.1, and which are not related to the phenotypes. With these large-scale genotypes, we empirically estimated the genetic relationship matrix [27], which was then used as **Φ** in the proposed methods. The empirical type-1 error rates were calculated from 10,000 replicates at the 0.005, 0.01, and 0.05 significance levels. Table 3 shows that the empirical type-1 error rates for MFQLS are approximately equal to the nominal significance levels in the presence of the population substructure. Figures 4 and 5 respectively show QQ plots from results for quantitative and dichotomous phenotypes when F_ST_ was assumed to be 0.01 and *ρ* was 0.2. The QQ plots showed that the statistical validities for both dichotomous and quantitative phenotypes were preserved at various significance levels.

**Table 3:** **Empirical type-I error estimates in the presence of population substructure**

				***α***		
**TYPE**	**F**_**ST**_	***Q***	***D*** **'**	**0.005**	**0.01**	**0.05**
Quantitative	0.01	2	0	0.0048	0.0098	0.0546
		2	0.5	0.0066	0.0105	0.0513
		5	0	0.0046	0.0098	0.0521
		5	0.5	0.0058	0.0105	0.0534
	0.05	2	0	0.0054	0.0094	0.0514
		2	0.5	0.0050	0.0108	0.0521
		5	0	0.0057	0.0094	0.0509
		5	0.5	0.0046	0.0094	0.0496
Dichotomous	0.01	2	0	0.0050	0.0107	0.0488
		2	0.5	0.0039	0.0082	0.0472
		5	0	0.0059	0.0108	0.0499
		5	0.5	0.0045	0.0089	0.0465
	0.05	2	0	0.0065	0.0125	0.0529
		2	0.5	0.0049	0.0108	0.0477
		5	0	0.0053	0.0115	0.0525
		5	0.5	0.0046	0.0093	0.0480

The empirical type-I errors were estimated using 10,000 replicates at several significance levels. We assumed that the number of markers is two, and that their minor allele frequencies were generated as *U*(0.1, 0.5). The phenotypic correlations were assumed to be 0.2.

The empirical power estimates for quantitative and dichotomous phenotypes are shown in Tables 4 and 5. The empirical power estimates were calculated from 2,000 replicates and the nominal significance levels were assumed to be 0.001 and 0.01 for quantitative and dichotomous phenotypes, respectively. The empirical power estimates for the proposed method were compared with those of MMASTOR and MFBAT for quantitative phenotypes, and with those of MMQLS and MFBAT for dichotomous phenotypes. The results showed that our method is always the most efficient, followed by MMASTOR for quantitative phenotypes and by MMFBAT for dichotomous phenotypes; this was also the case in the absence of population substructure. In particular, a greater reduction in power was observed along with the larger FST.

**Table 4:** **Empirical power estimates for quantitative phenotypes in the presence of population substructure**

***FST***	***ρ***	***Q***	***D*** **'**	**MMASTOR**	**MFBAT**	**MFQLS**
0.01	0.2	2	0	0.5020	0.1935	0.5680
		2	0.5	0.6860	0.3530	0.7570
		5	0	0.7380	0.3610	0.7965
		5	0.5	0.9065	0.6430	0.9180
	0.5	2	0	0.4765	0.1630	0.5300
		2	0.5	0.6710	0.3365	0.7390
		5	0	0.6820	0.2990	0.6975
		5	0.5	0.8450	0.5057	0.8600
0.05	0.2	2	0	0.4880	0.1925	0.5330
		2	0.5	0.6550	0.3250	0.6925
		5	0	0.7210	0.3465	0.7375
		5	0.5	0.8765	0.6430	0.8885
	0.5	2	0	0.4555	0.1620	0.4830
		2	0.5	0.6335	0.3150	0.6745
		5	0	0.6525	0.2995	0.6570
		5	0.5	0.8160	0.4850	0.8190

The empirical power was estimated using 2,000 replicates at the 0.005 significance level. We assumed that the number of markers was two, and that their minor allele frequencies were generated as *U*(0.1, 0.5).

**Table 5:** **Empirical power estimates for dichotomous phenotypes in the presence of population substructure**

***FST***	***ρ***	***Q***	***D*** **'**	**MMQLS**	**MFBAT**	**MFQLS**
0.01	0.2	2	0	0.2075	0.0565	0.2350
		2	0.5	0.3365	0.1135	0.3795
		5	0	0.3455	0.0975	0.3825
		5	0.5	0.6025	0.2330	0.6455
	0.5	2	0	0.1830	0.0545	0.2140
		2	0.5	0.2900	0.1165	0.3120
		5	0	0.2855	0.0910	0.3240
		5	0.5	0.5345	0.2200	0.5965
0.05	0.2	2	0	0.1975	0.0575	0.2300
		2	0.5	0.2840	0.0995	0.3210
		5	0	0.2990	0.0915	0.3335
		5	0.5	0.5405	0.2140	0.5860
	0.5	2	0	0.1680	0.0595	0.2065
		2	0.5	0.2605	0.1095	0.2930
		5	0	0.2620	0.0910	0.3025
		5	0.5	0.4835	0.1800	0.5370

The empirical power was estimated using 2,000 replicates at the 0.005 significance level. We assumed that the number of markers was 2, and that their minor allele frequencies were 0.2.

### Applications to a genome-wide association in the HTK cohort

The HTK cohort which consisted of families ascertained with healthy twins was initiated to identify genetic variation responsible for complex traits and the role of the environment in the etiology of complex diseases. HTK cohort consists of 2,473 individuals including 900 monozygotic (MZ) twins and 234 dizygotic (DZ) twins. In particular, MZ twins have same genotypes, and a single individual from each twin was randomly selected for genotyping. 1861 individuals were genotyped with Affymetrix Genome-Wide Human SNP array 6.0. We discarded SNPs with p-values for Hardy–Weinberg equilibrium (HWE) less than 10^–5^ or MAF less than 0.01, leaving 516,610 SNPs for subsequent analysis. The proportion of genotypes identical between individuals in each family was calculated and individuals with inconsistency between the genetic and reported relationship (n = 58) were excluded. At the same time, individuals with coding error about type of twin status were excluded, and in total genotypes for 1801 individuals were used for analysis.

The body mass index (BMI) is defined as individuals’ body mass divided by the square of their height and the waist-hip ratio (WHR) is the ratio of the circumference of the waist to that of the hips. The triglyceride (TG) is an ester derived from glycerol and three fatty acids, and we took a logarithm to TG. With these three phenotypes we performed joint analysis to identify the disease susceptibility loci for obesity-related phenotypes. Age and sex were included as covariates for the linear mixed model and BLUP was utilized as offset for MFQLS. The number of individuals with missing phenotypes for BMI, WHR, and TG were 4, 1, and 28, respectively, and their tijq were assumed to be 0. For comparison, EMMAX [26] based on linear mixed model was separately applied for each phenotype and covariates used for MFQLS were also included as those for EMMAX. We calculate genetic relationship matrix with common SNPs and they were used as variance-covariance matrix for EMMAX to adjust the population substructure.

**Figure 6:**
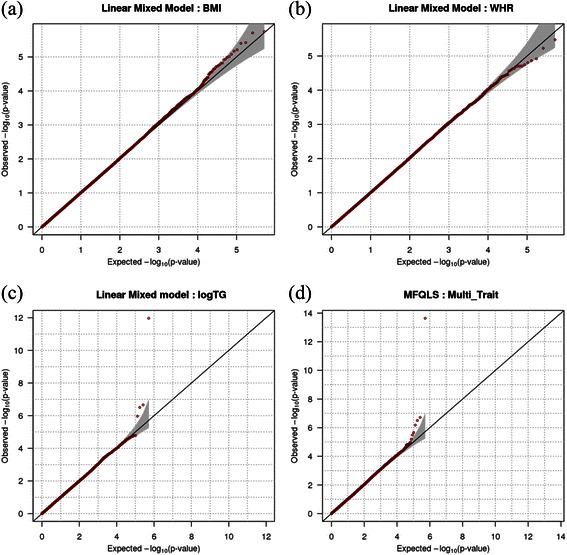
**QQ-plots for the results from the genome-wide association study. (a)** BMI, **(b)** WHR and **(c)** logTG was analyzed with EMMAX based on Linear Mixed Model. **(d)** BMI, WHR and logTG were jointly analyzed using MFQLS.

**Figure 7:**
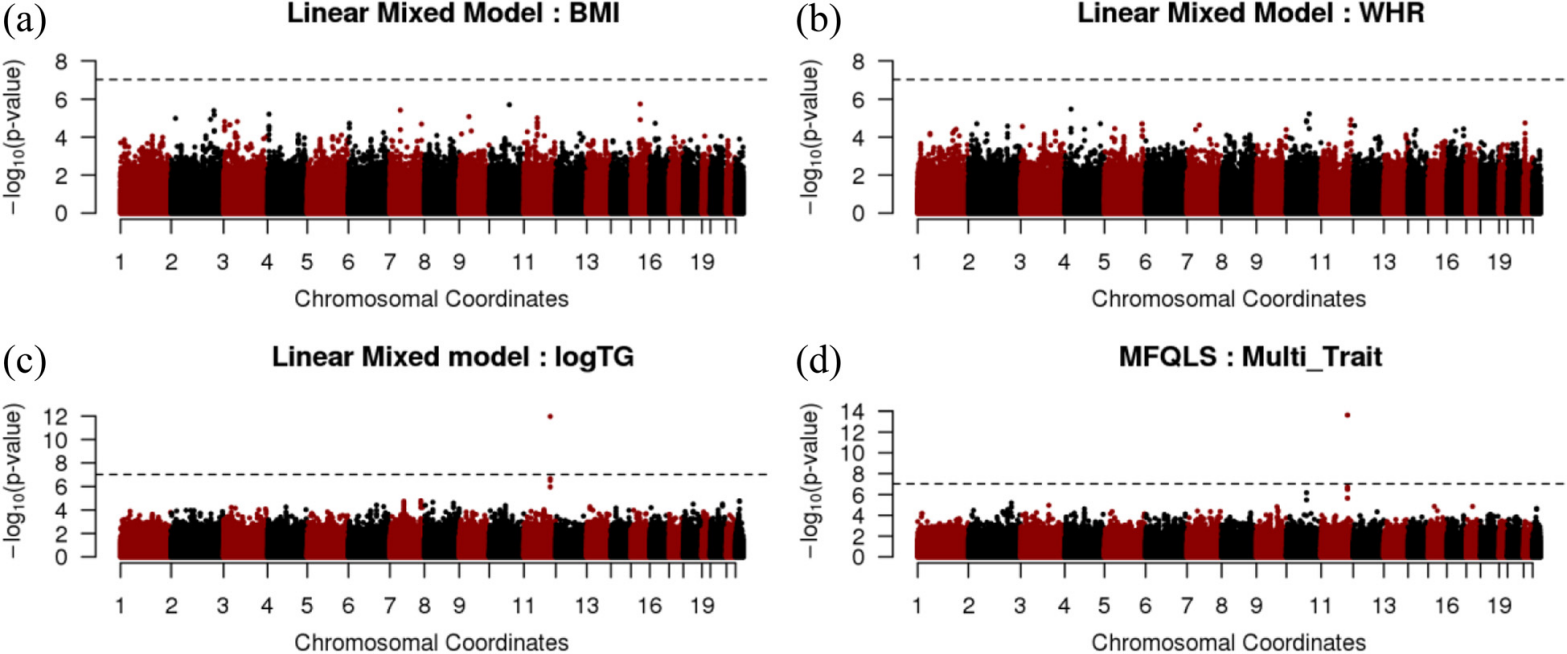
**Manhattan-plots for the results from the genome-wide association study. (a)** BMI, **(b)** WHR and **(c)** logTG was analyzed with EMMAX based on Linear Mixed Model. **(d)** BMI, WHR and logTG were jointly analyzed using MFQLS.

The QQ plots in Figure 6 show that the results for the EMMAX and MFQLS preserve the nominal significance level, and Manhattan plots in Figure 7 demonstrate that the results from MFQLS are more significant than the results from EMMAX. Genome-wide significance level with Bonferroni correction is 9.68 × 10^–8^ and Table 6 shows the results for SNPs of which p-values were less than 5 × 10^–7^ for EMMAX or MFQLS. rs651821 is an unique genome-wide significant result and the p-value of rs651821 derived by MFQLS was markedly less than those derived by EMMAX. P-values of rs17119975 and rs4417316 were larger than the significance level by Bonferroni correction but they are still expected to be promising candidate disease susceptible loci. In particular, the genetic positions of these three SNPs were similar, and we checked the linkage disequilibrium between theses SNPs with pairwise r^2^ from the Chinese and Japanese data in the HapMap Release 3. rs17119975 and rs4417316 were in linkage disequilibrium with r^2^ = 0.823, but r^2^ between rs651821 and the others are less than 0.01. Small p-values of rs17119975 and rs4417316 may be generated with the same genetic component even though both are located in different genes, and it should be noticed that the smallest p-value for rs17119975 and rs4417316 was found with MFQLS.

**Table 6:** **Significant results from genome-wide association study**

**SNP**	**CHR**	**POS**	**Gene**	**Minor allele**	**EMMAX**	**MFQLS**
rs651821	11	116167789	APOA5	C	1.075 × 10^-12^	2.295 × 10^-14^
rs17119975	11	116139767	BUD13	C	2.191 × 10^-7^	1.940 × 10^-7^
rs4417316	11	116157511	ZNF259	T	3.121 × 10^-7^	3.138 × 10^-7^

Based on those results, we conducted the gene-based analysis with MFQLS for those three genes. All SNPs in each gene were utilized for the joint analysis of multiple phenotypes and multiple genotypes. Single SNP is located in APOA5, and three SNPs are in BUD13 and ZNF259. The result for APOA5 is same as results for rs651821. Thus, our MQLS statistics assumes that *Q* = 3 and *M* = 1 for APOA5, and *Q* = 3 and *M* = 3 for BUD13 and ZNF259. Table 7 shows results from the MFQLS analyses, and we found that APOA5 and ZNF259 are genome-wide significant even though the genome-wide association analyses with *M* = 1 identified only a single genome-wide significant SNP. Therefore, the analyses of multiple genotypes provided more genome-wide significant results, and seem to be efficient strategy for association analysis.

**Table 7:** **Gene-based association analysis for APOA5, BUD13 and ZNF259**

**CHR**	**Gene**	**List of SNPs**	**P-value**
11	APOA5	rs651821	2.295 × 10^-14^
11	BUD13	rs11600380, rs17119975, rs1145208	1.331 × 10^-5^
11	ZNF259	rs4417316, rs6589566, rs603446	2.044 × 10^-9^

## Discussion

In this report, we have extended a score test based on the quasi-likelihood to joint analysis of multiple phenotypes and genotypes. The proposed method can be applied to dichotomous and quantitative phenotypes, and it is statistically valid even in the presence of population substructure. With extensive simulation studies, we found that the proposed method is statistically more efficient than existing methods. The genome-wide association analysis of the HTK cohort with *M* = 1 and *Q* = 3 required 13 minutes and 26 seconds. The pedigree structure does not affect the computational intensity and thus we can conclude that the proposed method is computationally efficient enough to complete genome-wide association analysis using a few thousand individuals within a few hours. The software for the proposed method is downloadable from http://healthstat.snu.ac.kr/software/mfqls/.

The proposed method is based on quasi-likelihood [31-33,44] and the relationship of the proposed method with the existing methods based on quasi-likelihood can be explained by different choices of **V** and **μ**. For instance, if *M* and *Q* are 1, the MASTOR statistic [44] used the phenotypic variance covariance matrix and BLUP for **V** and **μ**, respectively. If an identity matrix and prevalence are used, our method is equivalent to MQLS [31]. We empirically confirmed that, in retrospective analysis, the identity matrix was the most efficient choice for V and the most efficient choice of offset can be either BLUP or prevalence, depending the sampling schemes [31,33]. Our results for the joint analysis of multiple genotypes and phenotypes also yielded similar results. However, families for association analysis are often ascertained based on some family members and the choice of offset is not clear in such a scenario. This will be further investigated in our follow-up studies.

The proposed methods test the homogeneity of genotype distribution along the phenotypes, but this retrospective analysis is expected to be less efficient than the prospective analysis of random samples. However, it has recently been shown that power loss for retrospective analysis is often negligible [33], and the retrospective analysis can be preferred because of their flexibilities for genetic association analysis. For instance, first, the proposed method is robust to outliers and nonnormality of phenotypes. While the genetic heterogeneity between individuals can be adjusted with an estimated kinship coefficient matrix, nonnormality and outliers of phenotypes often lead to loss of validity or efficiency of the statistical inference [33]. In particular, when multiple samples are pooled, the heterogeneity of phenotypic distributions between samples requires stratified analysis, but the heterogeneity of genotypes between individuals may be controlled by using a genetic relationship matrix for retrospective analysis, which enables the direct analysis of the pooled sample. Second, the uncertainty of missing genotypes can be controlled using the proposed method. Missing genotypes are usually imputed based on linkage disequilibrium, and they were utilized for association analysis without consideration of the uncertainty of the imputed genotypes. However if the variation of the imputed genotypes is substantial and it is not considered for genetic association analysis, statistical inference can be invalidated. However the proposed method can consider the uncertainty of the imputed genotypes, and it enables the valid statistical inference in such a scenario.

Even though GWAS have successfully identified many genetic variants for diseases in the past decade, our experience has revealed that further investigation of the analysis strategies for reducing false negative findings is necessary. The significant results from our analysis with simulated data and real data for obesity indicated that joint analysis with multiple phenotypes and genotypes may provide a breakthrough in genetic association analysis.

## Conclusion

We proposed a new method for the joint analysis of multiple phenotypes and genotypes. There is no uniformly most powerful method for the joint analysis and the statistically most efficient method depends on the unknown disease model. The proposed method assumes that multiple genes have a causal effect on multiple phenotypes, and the genotype-phenotype models are multidimensional, multivariate analyses. In such a scenario, our method is expected to be an efficient strategy. The proposed method is implemented with C++ and the computationally efficient at the genome-wide scale. We feel the current methods open new ways to identify the disease susceptibility loci.
